# Recovery from life-threatening pelvic organ prolapse in an 80-year-old Japanese woman: a case report

**DOI:** 10.1002/ccr3.74

**Published:** 2014-05-23

**Authors:** Tomohiro Matsuo, Yasuyoshi Miyata, Kojiro Ohba, Yasushi Mochizuki, Hideki Sakai

**Affiliations:** Department of Urology and Renal Transplantation, Nagasaki University Hospital1-7-1 Sakamoto, Nagasaki, 852-8501, Japan

**Keywords:** Disseminated intravascular coagulation, pelvic organ prolapse recovery, septic shock, tension-free vaginal mesh operation

## Abstract

**Key Clinical Message:**

Pelvic organ prolapse (POP) is common among multiparous elderly women. POP related to obstructive anuria is very uncommon, but can be life-threatening if untreated. In this report, the patient survived from a septic shock with multidisciplinary treatment and was completely cured of POP after tension-free vaginal mesh repair.

## Introduction

Pelvic organ prolapse (POP) is common among multiparous elderly women. It is associated with minor lower urinary tract complications, including urinary tract infections and voiding difficulty [1]. POP related to obstructive anuria is very uncommon, but can be life-threatening if untreated [1]. Renal failure in the presence of POP may be due to the compression of the ureter by the uterine blood vessels. Furthermore, POP might cause obstruction of the lower ureters and disturb the bladder function [2]. In this report, we describe the case of a patient with POP who did not receive treatment for a long period of time. She survived from septic shock with multidisciplinary treatment and was completely cured of POP with a tension-free vaginal mesh (TVM) operation.

## Case Report

The patient was an 80-year-old Japanese woman who had previously given birth four times by vaginal deliveries. She had been experiencing voiding difficulty and perineal discomfort for 5 years and had urinary stress incontinence for 2 years. Furthermore, she had not been previously followed up for POP. She visited her previous doctor because of the onset of general fatigue and urinary retention. Bilateral moderate hydronephroureters with dilated calyxes were identified by computed tomography. Therefore, a urinary catheter was inserted, and ∼1100 mL of urine was collected. Although no urogynecologists were available, she was admitted to the hospital to recover from her general condition. Subsequently, she went into a state of shock due to low blood pressure on the day of admission. Although she was administered antibiotics and dopamine hydrochloride for 2 days, she was transferred to our hospital for treatment because of worsening renal dysfunction and state of shock.

**Figure 1:**
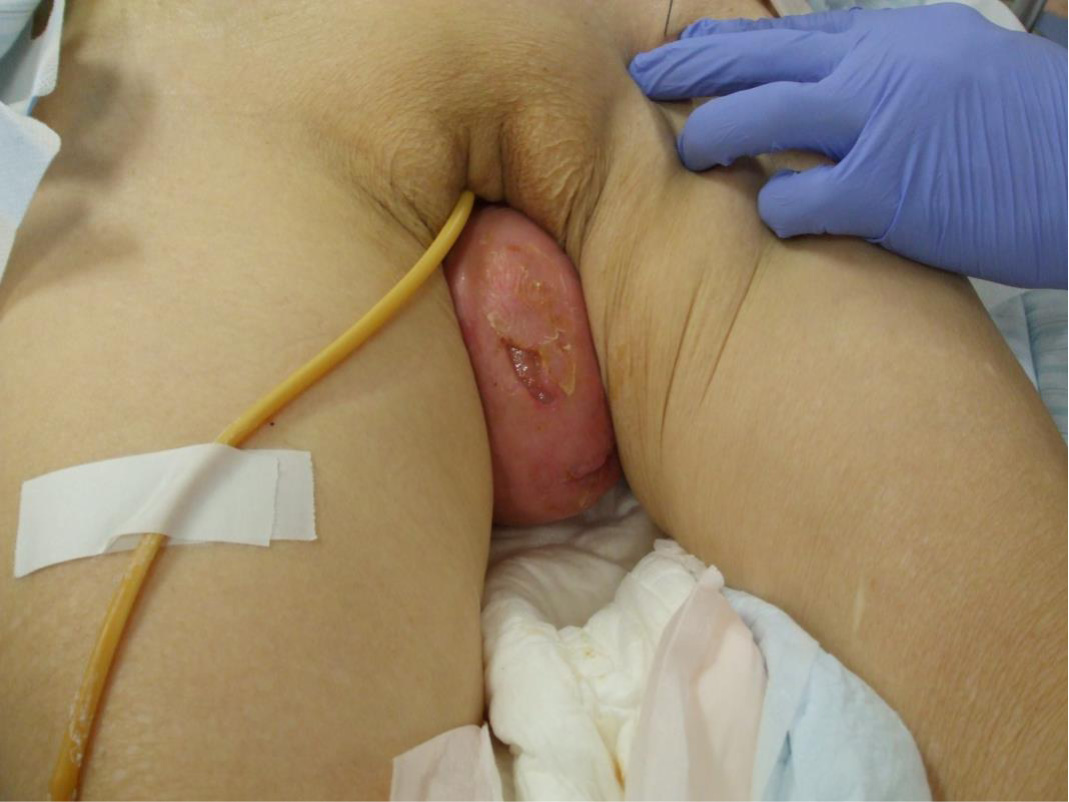
Total uterine prolapse was identified by gynecological examination.

A gynecological examination revealed total uterine prolapse (Fig.1). In addition to hydronephrosis caused by chronic voiding difficulty and excessive residual urine, she was presumed to have pyelonephritis, which resulted in septic shock. She had high levels of activities of daily living and was generally healthy before suffering from this disease. According to laboratory tests, she had a high level of fibrin degradation products (23.4 *μ*g/mL), high level of D-Dimer (9.8 *μ*g/mL), and a low platelet count (4.9 × 10^4^/*μ*L), which also resulted in disseminated intravascular coagulation (DIC). Both blood and urine cultures were positive for *Escherichia coli*. Ring pessary and bilateral ureteral stents were temporarily inserted for urine drainage. Furthermore, ceftriaxone, nafamostat mesilate, and thrombomodulin were used for treating pyelonephritis and DIC. Serum creatinine, a measure of renal function, increased to a maximum of 6.09 mg/dL before ureteral stent insertion and then decreased to 1.10 mg/dL after 15 days. She recovered from DIC and septic shock 8 days after this multidisciplinary treatment.

Her general condition completely improved 24 days later, and she was discharged from our hospital temporarily. We also suggested optional treatments (i.e., LeFort colpocleisis, vaginal hysterectomy with colporrhaphy, TVM procedure, and ring pessary insertion) to the patient, and she selected the TVM procedure without hysterectomy. She did not have any estrogen-related cancers or general diseases. Therefore, she was administered estrogen therapy for 3 months (i.e., 1 month before and 2 months after the operation) to prevent postoperative complications. She was readmitted to our hospital 3 months later, and an anterior and posterior TVM operation was performed. Surgery was performed under general anesthesia with the patient in a lithotomy position. We used the TVM surgical technique that has been previously described [3], with a few modifications. A monofilament polypropylene mesh (25 × 25 cm; Gynemesh PS; Ethicon, Somerville NJ) was cut into a shape similar to the shape used in the Prolift system (Ethicon). The initial procedure for anterior TVM was an anterior colpotomy. The next step included posterior TVM to correct the rectocele and prolapsed uterus. The mesh was precut and adjusted according to the type of correction required. We exfoliated around the uterine cervix and combined the anterior and posterior mesh with another 2 × 7 cm rectangular mesh strip that was rolled around the uterine cervix using a 2-0 monofilament nylon suture on both sides. Correct positioning was confirmed by traction on the exteriorized sling arms. The operation time was 147 min, and the blood loss was 200 mL. The bilateral ureteral stents were removed 3 days after the operation. The patient had a favorable course, with relief from voiding difficulty and hydronephrosis and no recurrence during for 2 years following the operation.

## Discussion

Symptomatic urinary tract infection is the most common infection among the elderly. Factors contributing to urinary tract infections in the elderly, especially women, include mechanical changes (i.e., reduction in bladder capacity, uninhibited contractions, decreased urinary flow rate, and post void residual urine) and hormone changes (i.e., low estrogen levels in postmenopausal women) [4].

Low estrogen levels also decrease vaginal muscle function, resulting in the weakening of ligaments holding the uterus, pelvic floor, and bladder, which ultimately leads to a prolapse of internal genitalia. Moreover, POP could also lead to various types of urinary disorders in patients. Therefore, POP could indirectly cause symptomatic febrile urinary infections. Gynecological examinations, during the presence or absence of POP, are important in cases of urinary tract infection and should be especially considered for elderly women with idiopathic fever [5].

When POP is the cause of urinary retention, temporary catheter placement is necessary not only as an emergency measure for POP itself but also for preventing urinary tract infections and preserving kidney function. This patient had already developed urinary tract infection because of chronic voiding difficulty caused by POP. She was in a state of acute renal failure, which had resulted from urinary tract infection and dehydration as prerenal factors and hydronephrosis as a post renal factor.

POP often causes hydronephrosis. Hydronephrosis is found in 5% of all patients with POP [6], and in 40% of patients with total uterine prolapse [7]. The obstruction may be caused by the compression of the ureters by the uterine vessels, the levator ani sling, or the uterine fundus [1,8]. The caudal displacement of the bladder trigone results in the compression of the ureters between the uterus and the medial borders of the genital hiatus [1,2]. Uterine prolapse may cause acute renal failure in some cases [2]. In previous reports of life-threatening cases, patients with POP and hydronephrosis or pyelonephritis requiring urgent treatment were mostly treated with ring pessary or hysterectomy (Table1). The patient described in this case report had sepsis and DIC resulting from acute pyelonephritis caused by POP and was treated by inserting bilateral ureteral stents to drain urine and pus. Her general condition was not good. Furthermore, we did not insert percutaneous nephrostomy tubes. She was completely cured of POP by multidisciplinary treatments and the TVM operation. To the best of our knowledge, no cases of patients with life-threatening POP, which resulted in DIC, have been previously reported. We consider our case to be very important because the uterus was preserved, renal dysfunction improved, and hydronephrosis was eliminated.

**Table 1:** Reports of life-threatening pelvic organ prolapse

Author	Patient age (years)	Complications	Therapy
Kang and Kim [1]	86	Anuria, UTI, ARF	Pessary insertion, antibiotics, nephrostomy
Begliomini and Begliomini [9]	59	Hydronephrosis, ARF	Endoscopic colposuspension
Sanai et al. [10]	64	Hydronephrosis, ARF	Pessary insertion, hemodialysis
Young et al. [8]	79	Urinary retention, hydronephrosis, acute pyelonephritis, septic shock	Pessary insertion, antibiotics
Young et al. [8]	79	ARF	Pessary insertion
Floyd et al. [11]	80	Hydronephrosis, ARF	Pessary insertion
MacKenzie et al. [12]	80	Urinary retention, hydronephrosis, anuria, ARF	Vaginal hysterectomy and pelvic floor repair
Chuang et al. [13]	67	UTI	Vaginal hysterectomy
Chuang et al. [13]	46	Hydronephrosis	None
Yanik et al. [2]	25	Dysuria, hydronephrosis, ARF	Vaginal hysterectomy
Micha et al. [14]	77	Hydronephrosis, ARF	Pessary insertion
Tsang et al. [15]	67	Hydronephrosis, ARF, perinephric and subcutaneous abscesses	Antibiotics, percutaneous drainage, vaginal hysterectomy
Matsuo et al. (This case)	80	Urinary retention, hydronephrosis, ARF, acute pyelonephritis septic shock, disseminated intravascular coagulation	Antibiotics, temporary pessary insertion, multidisciplinary treatment for disseminated intravascular coagulation, transvaginal mesh surgery

UTI, urinary tract infection; ARF, acute renal failure.

In this case, bilateral hydronephroureters were identified in the patient by computed tomography, and she had received antibiotic therapy at a previous hospital. Although we believe that she might have had total prolapse when she visited the previous hospital, she did not undergo any procedures for the treatment of POP. Therefore, POP should be considered as one of the differential diagnosis when treating patients with severe general illness.

## Conclusion

We have presented a case of a patient who was completely cured of POP following septic shock after TVM operation.

## Consent

Written informed consent was obtained from the patient for publishing this case report and accompanying images. A copy of the written consent is available for review by the Editor-in-Chief of this journal.
